# A Framework for Authentic Ethical Decision Making in the Face of Grand Challenges: A Lonerganian Gradation

**DOI:** 10.1007/s10551-021-04974-2

**Published:** 2021-10-21

**Authors:** Patricia Larres, Martin Kelly

**Affiliations:** Queen’s Management School, Riddel Hall, 185 Stranmillis Road, Belfast, BT9 5EE Northern Ireland

**Keywords:** Lonergan, Global crises, Transcendental precepts, Ethical decision making (EDM)

## Abstract

This paper contributes to the contemporary business ethics narrative by proposing an approach to corporate ethical decision making (EDM) which serves as an alternative to the imposition of codes and standards to address the ethical consequences of grand challenges, like COVID-19, which are impacting today’s society. Our alternative approach to EDM embraces the concept of reflexive thinking and ethical consciousness among the individual agents who collectively are the corporation and who make ethical decisions, often in isolation, removed from the collocated corporate setting. We draw on the teachings of the Canadian philosopher and theologian, Fr. Bernard Lonergan, to conceptualize an approach to EDM which focuses on the ethics of the corporate agent by nurturing the universal and invariant structure that is operational in all human beings. Embracing Lonergan’s dynamic cognitive structure of human knowing, and the structure of the human good, we advance a paradigm of EDM in business which emboldens authentic ethical thought, decision making, and action commensurate with virtuous living and germane to human flourishing. Lonergan’s philosophy guides us away from the imposition of over-arching corporate codes of ethics and inspires us, as individual agents, to attend to the data of our own consciousness in our ethical decision making. Such cognitional endowment leads us out of the ethics of the ‘timeless present’ (Islam and Greenwood in Journal of Business Ethics 170: 1–4, 2021) towards ethical authenticity in business, leaving us better placed to reflect upon and address the ethical issues emanating from grand challenges like COVID-19.

## Introduction

Today’s corporate world is faced with increasingly complex ethical decisions arising from unprecedented circumstances which include climate-induced natural disasters, a worldwide financial meltdown and a global health crisis. Corporations and regulatory bodies continue to meet grand challenges of this magnitude by scrutinizing and refining codes of ethics (Clarke & Dela Rama, 2006; Sikka, 2015). Yet, the acceptance of top-down, rules-based compliance ethics, in pursuit of a rational and universal basis for the exercise of human moral autonomy, pays scant attention to the ongoing negotiated quality of ethical agency and moral decision making within a corporate context. Moreover, imposing an ethical code on corporate players can have an antipodal effect on moral practice whereby agents offload their personal moral responsibility onto corporate ethical rules (Clegg et al., 2007). This “well-established approach…to mediate crises” (Sikka, 2015, p. 2) fails to recognize that “ethical responsibilities lie, not with the corporation, but with the individual agents, real persons, who form and function the corporation” (Miller, 2005, p. 220). This is particularly the case with respect to COVID-19, where adaptive responses to the pandemic have emphasized isolated behavior such as working from home and observing physical distancing protocols (Kniffin et al., 2021) so that agents representing the corporation engage in more introspection and remote decision making.

This paper makes a theoretical contribution to the contemporary business ethics narrative by proposing an approach to corporate ethical decision making (EDM) which serves as an alternative to the imposition of ethical codes. Our approach to EDM embraces the concept of reflexive thinking and ethical consciousness among the individual agents who collectively are the corporation. In so doing, it moves the narrative from “the ethics of…the timeless present” (Islam & Greenwood, 2021, p. 3) to one which captures the reality of transformational change within business and the wider society. This contribution is made on a number of levels. First, we answer the recent call by Islam and Greenwood (2021) to reconnect to the social in business ethics. By adopting a Lonerganian approach to EDM we move “beyond normative accounts based on established ethical frameworks (utilitarian calculation, rule compliance, etc.)” (Islam & Greenwood, 2021, p. 3), to imagine new forms of promoting and sustaining human flourishing within business organizations. Such an approach to ethics, we believe, enables a view of the future while simultaneously taking account of the present and the past. The ethical responsibility for meeting the needs of others, is grounded, not in some universal doctrine or codes of practice, but in the rational self-consciousness of subjects (i.e., human beings). Responsibility, requires us to look beyond our immediate horizon and imagine alternative viewpoints, enlarge our understanding of a situation and engender a willingness to act for the wider human good. Second, we diverge from conceptual frameworks which uphold EDM on the basis of a priori criteria and proffer an alternative which promotes the nurturing of the universal and invariant structure that is operational in all human beings. Built on the workings of the human mind, we present an epistemology which embraces the concept of reflexivity, a self-awareness that is characteristic of human consciousness, an attribute absent from other ethical models in the literature (Crossan et al., 2013). Business ethics is a product of our immanent and invariant operations of consciousness and so, drawing on Bernard Lonergan’s dynamic cognitive structure of human knowing, we argue that his transcendental method presents not only an explanation of cognitive activity but also fosters heightened self-awareness and reflexive knowledge: dimensions of intentional performance that enable deliberate participation in fulfilling ethical action. Third, we argue that self-appropriation, as propagated by Lonergan, adds a deeper appreciation and intensified awareness of the recursive cognitive activities inherent in EDM. Lonergan’s teachings, we believe, contribute to the development of an invariant EDM framework by which ethical imaginaries (Islam & Greenwood, 2021), namely a “disengaged view from somewhere” (Werhane, 1998, p. 90) which is self-critical and cognizant of the particularities of situations, can be concretely realized in business organizations.

As scholars, there is a moral imperative on us to critically reflect on EDM processes to better “guide business leaders, employees, and stakeholders with systematic, unbiased, and robust evidence on mechanisms with which to tackle the persistent global problems confounding us” (George et al., 2016, p. 1893). This moral responsibility is more to the fore than ever in the midst of a pandemic that uniquely embodies both the transient and devastating nature of a natural disaster together with the scope and protracted magnitude of a grand challenge. Consequently, we must be committed to pushing back the boundaries of our domain and reaching out to other disciplines to explore new ways of theorizing EDM that capture the dynamic and recursive nature of the process. The work of Canadian philosopher and theologian, Fr. Bernard Lonergan, helps us to achieve this objective. Drawing on his seminal writings, we transcend narrow traditional ethical methodologies to present a framework which guides us as individuals to discover in ourselves the dynamic structure of our own cognitional and moral being and the implication of this discovery for EDM within business. We go beyond Lonergan’s (1992) constellations on bias, as explored by Miller (2005), to demonstrate how ‘bias’ is an interference with inquiry and with the self-correcting questions and answers that constitute the development of the dynamic cognitive structure of human knowing (Byrne, 2016) and how objectivity in knowing facts is indispensable to objectivity in knowing and acting ethically. In these times of unprecedented social, economic, and moral turmoil, Lonergan’s ethics of personal and shared authenticity, with an emphasis on human agency, provide a clarification of the normative requirements necessary for ensuring fidelity to the process of ethical inquiry and decision making.

The starting point of a thematic Lonerganian decision-making process is not a set of standardized rules, routines, or norms, but the intelligent, reasonable, and responsible selves that those comprising the corporation should strive to become. Our contention is that Lonergan’s philosophical anthropology encourages a dynamic understanding of morals through personal and communal horizons whereby we seek answers to, and find space for, fundamental questions about human life. In so doing we experience new ways to collaborate and organize, innovate, and, as knowing subjects, become more authentic human beings. As agents within a corporation, functioning virtually or otherwise, there is a need to create a set of shared experiences, understandings, and judgments that reach out and reflect the best of our past as well as being receptive to new voices and new perspectives. We explore the potential for Lonergan’s work to speak to everyman by reaching out to the essence of human knowing and serving the intrinsic nature of the human good.

The paper proceeds as follows: in the first section, we offer a short critique of capitalism as a corporate model which has stifled virtuous intent and acknowledge the opportunity for change emerging from the global pandemic. Thereafter, we advance a more human paradigm of EDM which focuses on the pivotal role played by virtue ethics in realizing both individual and collective flourishing. In the next two sections we conceptualize our EDM framework by embracing Bernard Lonergan’s philosophical anthropology and, in particular, his dynamic cognitive structure of human knowing and the human good which nurtures innovative thinking, questioning, and intervening to propagate a virtuous cooperative society. Finally, we demonstrate how Lonerganian ideas shape an organizational EDM framework which encourages individual self-appropriation of cognitive operations. In so doing, we seek to encourage ongoing critical enquiry and reflexive thinking to promote a culture of individual responsibility in decision making commensurate with today’s business environment.

## Towards Virtuousness and Human Flourishing in Ethical Decision Making

Lonergan’s contribution to the business ethics literature is best contextualized with reference to the excesses of capitalism and the lamentable toll which the policy model has taken on society’s virtues (Moore, 2005). In 1981, Alasdair MacIntyre (2013) warned that the capitalist’s dominant pursuit of external goods under assumptions of short-termism, self-interest and wealth maximization, ensures that “the concept of virtues might suffer first attrition and then perhaps something near total effacement” (MacIntyre, 2013, p. 228). A perfect exemplar of MacIntyre’s portent was the financial services sector prior to the 2008 global financial crisis during which time pecuniary gain was the sole measure of success and money became the goal rather than the tool of the sector (Ballantine et al., 2018). However, events pertaining to the recent pandemic have cast a long shadow over the capitalist model’s trajectory, and there is no knowing how MacIntyre’s warning will play out in the face of the economic and social uncertainty visited on the modern capitalist economy by COVID-19. At the very least, some economic and social reformatory good must emerge from what is first and foremost a global health crisis. There may be “an opportunity to use this crisis as a way to understand how to do capitalism differently” (Mazzucato, 2020). Crises often present the opportunity “to make deep-seated reforms. There is solid evidence from history that emergencies like COVID-19 offer valuable windows of opportunity to make big policy shifts, as established protocols break down and resistance to change is stifled” (Tony Blair Institute for Global Change, 2020, p. 6). The pandemic crisis has undoubtedly highlighted “the mutual interdependence of business and society” **(**Tony Blair Institute for Global Change, 2020, p. 19) and delivered a shared challenge to us all. The realization that this is something that affects everyone is likely to “raise people’s expectation of businesses [as] being more socially responsible” (He & Harris, 2020, p. 177).

At the micro level, COVID-19 has disrupted normal work routines and hastened the “acceleration of trends that were already underway involving the migration of work to online or virtual environments” (Kniffin et al., 2021, p. 65) so that, for many, social distancing and remoteness approaching isolation have replaced collocated settings. “The challenges for individuals working in this manner are clear: more people will need to learn to work in ways far different than how previous generations worked” (Kniffin et al., 2021, p. 74). Foremost among these new work experiences is independent decision making and a focus on the self-aware agent. Consequently, there is a pressing need to reinvigorate a bottom-up approach to EDM which recognizes that ethical responsibility must ultimately rest with the individual agents who comprise the corporation rather than with the corporation itself (Miller, 2005). To deliver on a social level and achieve a humanistic business ethos wherein corporations function as communities of decision-making human beings, virtual, or otherwise, we must turn our attention away from corporate capitalism to the common good theory of the firm and the role of virtue ethics (Melé, 2012).

Central to attaining common good for a corporation, and subsequently for society as a whole, is the cultivation of and obedience to one’s internalized virtues (Sison & Fontrodona, 2012). If virtues “are not internalized in actors as normative resources or dispositions, then no institutional procedure can evoke them” (Offe, 2012, p. 667). Virtues, according to the Aristotelian conceptualization, highlight the characteristics of the actor as indispensable normative qualities in EDM (Samsonova-Taddei & Siddiqui, 2016). Not only are they an essential facet of the individual, they are also the excellences that a society requires (Solomon, 1992). To this end, virtue ethics has been promoted by many ethicists over the years as a basis for business ethics (see for example: Beadle & Moore, 2006; Koehn, 1995). What is required, therefore, is a decision-making framework which is sympathetic to the exercise of virtues inside practices. Good judgment in EDM emanates from good traits of character, not from adhering to a formalized protocol (MacIntyre, 2013). To appreciate the tendencies which will help us to do the right thing, we must engage in meaningful ethical reflection. Without ethical reflection, we become self-enclosed and unwilling to challenge familiar and habitual actions derived from uncritical acceptance of rules and standards. This critical reflection can only be attained when reflexive thinking becomes embedded as a guide to ethical judgment. Nowhere has this issue been addressed more efficaciously than in the writings of Bernard Lonergan who challenges us to raise questions about both the cognitive process of EDM and moral agency within business organizations.

As a Jesuit priest confronted by the socio-economic and political vagaries which dominated the twentieth century, Lonergan’s philosophy and theological method, together with his dedication to social issues, steered him along a methodological path which rejected the extremes of atheistic communism on the one hand and the self-serving, avaricious nature of capitalism on the other (Melchin, 2012). Lonergan’s disaffection and disillusionment with twentieth century excesses and abuses compelled him to seek an alternative exemplar of authentic living which would transcend the moral confusion of the time. He was concerned that the problematic and uneven course chartered by modernity had resulted in citizens gradually losing confidence in their capacity to achieve objective knowledge, to make responsible moral choices and to lead lives of authenticity and truth. Today, when many in society have lost hope as we are catapulted from one global crisis to another, Lonergan’s message resonates. His teachings, particularly around critical inquiry and self-appropriation, provide respite and succor by establishing norms for authentic ethical thought, decision making, and action. By encouraging us to appreciate the pivotal role played by ethical reflection as a constituent element of human living, Lonergan’s teachings allow for development of the self and the social order and ultimately towards human flourishing. Engaging with Lonerganian philosophy can add conceptual depth to current discussions not only on the pandemic but also on issues of corporate social and environmental sustainability. Such “pragmatic experimentation” (Wicks & Freeman, 1998, p. 124) provides a critical role for ethics in humanizing business organizations and helping people attain a “thorough and ready access to their own fulfillment” (Vatican, 1965, para. 26).

Through a Lonerganian lens we can explore a process which guides us to discover in ourselves the dynamic structure of our own cognitional and moral being and the implication of this for EDM in business. In so doing, we come to realize that business and ethics are not fated to be in a constant state of conflict. Engaging in authentic EDM necessitates deliberation on how courses of action will contribute to the betterment of the self and to the attainment of the common good. Lonergan chaperones us in this endeavor and helps deliver an EDM framework to address the challenges present in today’s corporate environment.

There is, of course, a large number of normative and descriptive EDM frameworks already pervading the business ethics literature (see for example: Dedeke, 2015; Hunt & Vitell, 1986; Jones, 1991; Kish-Gephart et al., 2010; Treviňo, 1986). So why do we need Lonergan? The extant frameworks, many of which are built on Rest’s (1986) four-stage process for decision making, tend to encompass the act theories of consequentialism and deontology (Crossan et al., 2013) and as such, leave business ethics firmly positioned in the timeless present. Within the literature, EDM models divide the postulated influences on an individual’s decision-making behavior into two broad categories, namely individual factors and situational factors (see, for example, Jones, 1991; Trevino, 1986). Our Lonerganian framework, in contrast, embodies the person, providing perspective beyond our immediate horizon to imagine alternative viewpoints, extend our understanding of a situation and engender a willingness to act for the wider human good. We offer a path to ethical authenticity, that is to say, going beyond oneself and applying the transcendental precepts of being attentive, intelligent, reasonable, and responsible. These precepts elucidate the essential nature of being human and present the guiding principles of human flourishing. In so doing, we present a novel EDM perspective which proffers a new vantage point on developing an ethical business culture and organizational strategy that encourages everyone in the organization to engage in self-appropriation of the operations of consciousness to meet society’s grand challenges.

## Ethical Decision Making Founded on Lonergan’s Dynamic Cognitive Structure of Human Knowing

Human knowing originates in the “pure desire to know,” in “the inquiring and critical spirit of man” (Lonergan, 1992, p. 348). There is no human knowledge, no real answer, without a prior question. Lonergan emphasizes that human knowing is both conscious and intentional, a consciousness and intentionality set in motion by the underlying dynamism of the unrestricted desire to know. With intentionality, objects are made present to the subject, and with consciousness, the subject becomes present to him/herself in cognitive operations (Lonergan, 1972). After all, “once we have awareness of the… mental models operative in our thinking, we can begin exploring means by which to correct them. It is that cognition that engenders responsibility” (Werhane et al., 2011, p. 114). As conscious subject, the individual is present to him/herself in many acts, not as the object of some “inward look” (Lonergan, 1992, p. 299), not as the subject-as-object, but in conscious self-presence. The individual moves from accepting commonplace rules and routines to proactive inquiry about the values that underpin and transcend such rules and routines. For both Lonergan and Aristotle, determining an ethical truth requires embracing concrete presentations and inspection of real contents.

At the core of Lonergan’s philosophy is the dynamic structure of human knowing, a cognitional activity of a particular knowing subject. Human beings have an unrestricted desire to know and, when properly developed, that spirit of inquiry will draw each person through a pattern of questioning which leads to correct understanding and, ultimately, to forming judgments. Lonergan reminds us of Aristotle’s basic assertion that “all men by nature, desire to know” (Aristotle, 1984, p. 1552) by stating that “deep within us all, emergent when the noise of other appetites is stilled, there is a drive to know, to understand, to see why, to discover the reason, to find the cause, to explain” (Lonergan, 1992, p. 28). The current pandemic has provided an opportunity to quench this thirst for knowledge insofar as the widespread restrictions placed on many everyday activities have made space in our hitherto busy lives for introspection. We have an opportunity to ask deeper questions about what we want as a society, questions that, in the midst of complacency and uncertainty, too often remain hidden beneath the surface. Central to Lonergan’s philosophy is the invitation to pay attention to our acts of insight. Lonergan rejects the idea that factual objectivity consists of an accurate representation, namely matching an idea formed in the mind with how things actually stand in the external real world. Instead, he postulates “that genuine objectivity results from authentic subjectivity – that is, faithfulness in answering all the questions posed about whether or not things really are so” (Byrne, 2016, p. 7). Objectivity then cannot be separated from the knowing and deliberating subject. Lonergan’s concern, then, is to help us discover, in-built in the activity of our own consciousness, the method by which we can reach truth and genuine value. Insights come as answers to questions, releasing the persistent and almost unbearable tension of inquiry that yearns to understand what we do not yet understand (Byrne, 2016). Lonergan’s distinctive contribution comes from tracing questions back to their originating and invariant sources in the two most basic human desires, namely “our unrestricted desire to know the whole of reality, and our equally unrestricted desire to discover and actualize whatever is genuinely good” (McCarthy, 2016, p. 141). These desires (or transcendental notions) are culturally invariant because of their unrestricted orientations and because all specific cultural contents are constructed in response to them. Lonergan illuminates how we can achieve self-transcendence through what he calls the transcendental method.

**Fig. 1:**
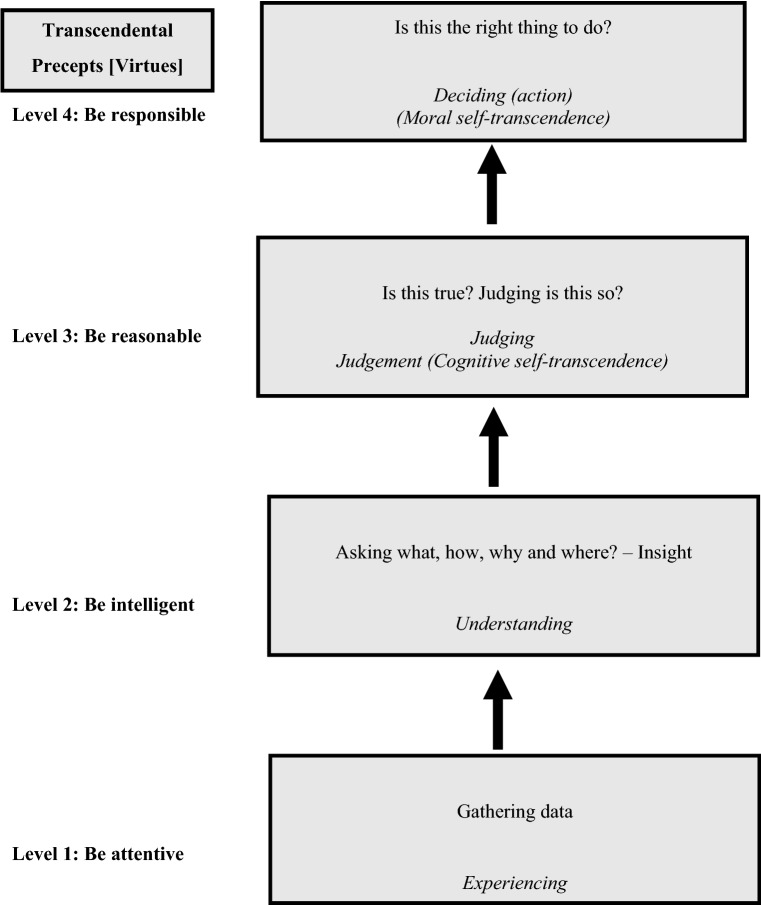
A Lonerganian ethical decision-making framework

As shown in our Lonerganian EDM framework depicted in Fig. 1, it is through the transcendental method that, in coming to know something, we are attentive to our experiences (level 1); we ask questions about our experiences and receive insight (level 2); we follow up by reflecting and determining whether our insights are correct (level 3); and finally we take decisions and act (level 4). The nexus of the transcendental method is what Lonergan refers to as ‘self-appropriation’ (Byrne, 2016). Insight momentarily breaks the tension of inquiry by discerning a possible solution to the problem or question in which the investigating subject is absorbed. It is through personal appropriation of what is already going on in consciousness that we are on the way to discovering what we are actually doing when pursuing knowledge. Experiencing precedes knowing, it does not constitute knowing. It provides the data of the first dimension or level of knowing. This first level is the world experienced through the senses. From birth, we begin experiencing in what Lonergan calls “the world of immediacy” (Lonergan, 1972, p. 28). But this is only the first part of what constitutes full human knowing. Questions for intelligence drive the search for making sense of the data: What is it? How does it work? Subsequent to developing an insight and formulating an understanding of it, the desire for precise understanding compels us to ask further questions for reflection. Is it so? Are you certain?

At the outset, concern rests with the orientation of the individual within the community. In the introduction to his book *Insight*, Lonergan adopts a first person inquiry approach which he calls self-appropriation and describes how his concern is not with the existence of knowledge or with what is known, but with the structure of knowing and with the personal appropriation of the dynamic and recurrent operative structure of cognitional activity as a method of coming to terms with oneself as a knower (Byrne, 2016). Moreover, Lonergan challenges us to be attentive to ourselves as operating subjects and to appropriate these internal operative norms of our own consciousness. At its root, self-appropriation consists of the transcendental notions that both enable us and require us to advance in understanding, to judge truthfully and to respond to values.

Lonergan is clear and assertive as to why this is important. “Thoroughly understand what it is to understand, and not only will you understand the broad lines of all there is to be understood but also you will possess a fixed base, an invariant pattern, opening upon all further developments of understanding” (Lonergan, 1992, p. 22). Lonergan shows us how an appropriation of engagement with these cognitive operations opens up a vast field of learning about self and about engagement with the wider world. “We have to perform the activities and go through the routines that will bring to explicit consciousness the dynamic aspect of the process of knowing” (Lonergan, 1990, p. 60). The drive towards value draws the subject through the process of self-appropriation as shown in Fig. 1. This fourth level of the EDM framework is distinguished by questions for deliberation which subsume the previous cognitional levels. Assessing value and choosing our actions occur at this level of rational self-consciousness. Such deliberation results in a judgment on a higher level that incorporates a value perception of what is truly good. Through self-appropriation, we pay attention to the operations of ethical reflection rather than the objects of reflection. This results in moral self-transcendence or authenticity in which the human person goes beyond knowing (Melchin, 2005). This is achieved in the mode of interiority where we attend to and encourage our own reflections on values and social goods in the context of everyday living. Authenticity is characterized by what Lonergan calls four transcendental precepts: be attentive (to the data); be intelligent (in inquiry); be reasonable (in making judgments); and be responsible (in making decisions and taking action). These transcendental precepts “constitute the very dynamism of our conscious intending, promoting us from mere experiencing to understanding, from mere understanding towards truth and reality, from factual knowledge to responsible action” (Lonergan, 1972, p. 12). Therefore, we can pursue the development of ethical engagement by adverting to how we actually experience, understand, judge, and decide to enact what is truly responsible. To be clear, self-appropriation is the opposite of a rationalizing ideology in that the purpose of an ideology, in its diverse forms, is to justify bias and halt the thrust of self-transcendence.

Human beings are often inattentive, unintelligent, unreasonable, and irresponsible in their actions leading to ‘unauthenticity.’ Lonergan’s transcendental precepts are imperative in that they point to what ought to be. For example, we experience data, so we ought to be open to experience (i.e., be attentive). By following the dictates of our own responsible consciousness, namely adhering to the transcendental precepts, we can become authentic knowers and deciders of value. By ignoring issues, turning a blind eye, refusing to inquire further, and so on, we diminish our authenticity. How often do we see business managers ignoring the wider social and environmental impacts of their operations? Suppressing discussion or dissent, lying, or obscuring the evidence is unreasonable and destroys authenticity. We can decide to share or, alternatively, we can wrap ourselves in self-interest. Moreover, we can choose the satisfactions of self-interest and selfish egoism, as opposed to the good of cooperation and order (Cronin, 2006).

The movement upwards in Fig. 1 is propelled by the dynamic of the question which drives the subject from level to level so that he/she moves “from mere experiencing to understanding, from mere understanding to truth and reality, from factual knowledge to responsible action” (Lonergan, 1972, p. 12). Moral knowing is not a singular act, but a chain of operations which, working together, propels us towards knowledge and the actuation of value. The characteristic form of each of the cognitive operations is that of questioning. The combined operations of sensing and understanding require an additional operation, namely, judging. In contrast to the process of reflection in judgments of fact, the process of deliberation does not end with a judgment. Instead, deliberation finds its realization in decision.

It is this dynamic structure of our cognitive activity which defines what we do when we know and our moral character when we decide. The norms of our conscious intentionality lead this process by demanding that our knowing be attentive, intelligible, objective, and responsible (Morin & Richards, 2010). These interrelated operations involved in knowing and doing are both intentional and conscious. They are intentional in that they make objects psychologically present to the subject. They are also conscious in that they make the performing subject present to him/herself, not as an object, but as a subject. It is the unifying character of the cognitive structure which makes it compelling. In other words, the cognitive process transcends all fields of study. The acquisition of personal knowledge in pursuit of ethical intentionality offers an antidote to codes in that codes impose a perceived truth which necessitates a kind of distancing from the complicated contexts in which judgments are made and reflected upon (Painter-Morland, 2008). Complying with codes can result in decisions which are overly simplistic or worse, forced to fit a rule (Smith & Dubbink, 2011). Such an outside-in approach to ethics may lead to problems pertaining to indeterminacy and generalism (Dancy, 2004). This imposed ethic, more often than not a consequence of legal or professional conformity, takes on the guise of regulatory posturing. In other words, it is an external gesture behind which promotion of self-interest, at the expense of the good in common, continues unabated. This scenario depicts the hitherto box-ticking reality of imposed business ethics in a capitalist world where a deficit in virtuousness coupled with an absence of communal cohesion to counteract predatory notions and facilitate participation in the goods of society, offers up a contemporary manifestation of Lonergan’s anguish and despair at humanity’s downward spiral.

As shown in Fig. 1, it is through cognitive and moral self-transcendence (namely reaching judgment and choosing a value that existed beyond ourselves and independent of our own activity) that we achieve the goal of attending to our conscious intentionality. By making daily choices about what is right or wrong, we have the opportunity to develop practical wisdom and internalize virtues (Mintz, 2010) such as attentiveness, intelligence, reason, and responsibility that, in turn, assist us in making informed ethical decisions. As human beings, we are born into a community of shared meanings and values, and it is from such a common pool of shared meanings that we progress and provide for our own well-being by contributing to the common good. In this respect, Lonergan’s philosophy emphasizes the dual dialectic of the subject and the community (Ogbonnaya, 2013).

Business organizations are communities of people (Solomon, 2004). This still holds true during the increased remote and virtual work practices resulting from COVID-19 restrictions. A business entity, in Lonergan’s world, is constituted by common meaning and motivated by value (Cronin, 2006). Moreover, “his view goes against the tendency to reify it – to forget that it is the product of human endeavor – and insists that it exists because certain events take place within the subjectivity and intersubjectivity of several people” (Komonchak, 2008, p. 168). Lonergan envisioned a corporation that would have, as its norm, the cognitive structure of human knowing. He viewed this as an open community of collaboration, underpinned by free, attentive, intelligent, reasoned and responsible inquiry and engagement. In a communal context, moral judgment, notwithstanding other cognitive abilities it may require, must involve the enlarged thought. Consequently, our capacity to make up our mind becomes the fulcrum “in an anticipated communication with others with whom… [we know we] must finally come to some agreement” (Arendt, 1961, p. 221). Such a view “emphasizes both individuals and the whole, making explicit the uniqueness, conscience, free will, dignity, and openness to self-realization of each one who forms the community” (Melé, 2012, p. 97). It is shared experience, common understanding, shared truths, and values that constitute the core of a community of persons relating to one another (Cronin, 2006). We, as humans, operate on the four levels of consciousness with the transcendental precepts operating at each level. By extension, we identify the community as common experience, shared understanding, agreed truths and values pertaining to a common way of life (Cronin, 2006). For Lonergan (1974), the proper function of a community’s culture is to secure and communicate the meaning and value of a society’s way of life. When a culture is functioning properly, it not only infuses social cooperation with meaning and purpose, but it also criticizes and revises social practices in response to new circumstances as they arise both internally and externally. The challenge posed by a national or international crisis requires finding methods that will sustain “tireless individual and collective efforts to preserve, promote, and augment what is good and to correct or repair what is harmful and broken to give correlative expression, socially, politically, and globally to the transcendental notion of value” (McCarthy, 2016, pp. 30–31). Progress “proceeds from originating value, from subjects being their true selves by observing the transcendental precepts” (Lonergan, 1972, p. 53). The Lonerganian EDM framework in Fig. 1 is a dynamic, self-assembling pattern of the knowing operations, fully conscious and spontaneous in each of us. When the flow of our dynamic cognitive structure is obstructed by conscious or unconscious blocking of questions that would generate unwelcome answers, our judgments and decision making become biased and unreliable (Snedden, 2017). Bias is anything that prevents the dynamism of our minds from attending to experience, seeking to understand, and making an evidenced based judgment. Individual bias (Lonergan, 1992) results in the individual good taking precedence over the good of others, ultimately leading to the erosion of the common good (Ogbonnaya, 2013), while group bias, according to Lonergan, occurs when the group’s interest within the organization is protected to the detriment of other groups. Uncritical loyalty to bias-ridden groups can impede longer-term progress in business organizations by clinging to position and power in contrast to seeking value as a greater good. We can see evidence of this during the current pandemic where key political leaders have insisted on downplaying the severity of COVID-19. Another example of group bias is the ongoing denial that healthy people, despite being asymptomatic, risk transmitting the virus. Those who have lost family members to the disease, or have lived with severe illness, are less likely to fall prey to this type of group bias because experience has already required them to ask questions. Ethical intentionality must incorporate genuine knowledge of the situation in which the ethical subject finds him/herself. “One cannot do good without knowing the facts, without knowing what is really possible, without knowing the probable consequences of one’s course of action” (Lonergan, 2004a, p. 37). Developing the capacity for good common sense judgments is, therefore, indispensable when becoming ethically responsible (Byrne, 2016). Judgments of facts give us the knowledge of the situations (i.e., what is going on?) that form the context for our ethical actions. Judgments of value (level 3) form the basis for our responsible ethical decisions and actions. At level 4 of the Lonerganian EDM framework (Fig. 1), one asks ‘shall I do it?’ This presupposes virtues such as attentiveness, intelligence, and reasonableness but requires a conscious act of deciding or choosing. Thereafter, action has to be taken. The ‘it’ in the question relates back to the content of judgment of value and reveals the dynamic relationship between acting and doing. In other words, action and choice are always dynamically related to prior acts of consciousness. There are times when common sense breaks down. In these instances, general bias can counter the evidence from theoretical knowledge and its representatives (i.e., ‘the experts’). The current pandemic reminds us of the ever-present dangers of general bias whereby many people dismiss the warnings from the scientific community regarding an invisible virus. We choose what is truly valuable provided we perform the prior cognitive activities (levels 1–3) in complete fidelity to the standards of our own ethical inquiring. However, “the process of deliberation and evaluation is not itself decisive, and so we experience our liberty as the active thrust of the subject terminating the process of deliberation by settling on one of the possible courses of actions” (Lonergan, 1972, p. 50).

Therefore, “choices are authentic when they choose judgments of ethical value that are produced by the cognitive structure operating in its full undistorted movement” (Byrne, 2016, p. 112). Failure to do so is often located in corrupt judgments which in turn can be traced back to the failure to ask and answer questions at the earlier levels (i.e., levels 1–2). Lonergan’s cognitional structure guides us to apprehend the dialectical nature of human knowledge that struggles between “bias and truth, inattention or insight, irresponsibility or responsibility” (Copeland, 1991, p. 38). The more we become attentive, intelligent, reasonable, and responsible, the more we become authentic and virtuous individuals. It is through habitually appropriating the transcendental precepts or virtues, namely be attentive, be intelligent, be reasonable and be responsible that we, as a community of human beings, come to learn and value the goodness of virtuous actions. “The true good, the objective value, is what is judged to be good by a person achieving self-transcendence and being authentic” (Lonergan, 2004b, p. 12). Lonergan’s cognitional structure demonstrates how virtuous living can be achieved through adherence to the transcendental precepts and a conscious intentionality to live authentic lives. The moral question emerges in knowing and doing. Essentially, we are morally obliged to not only act well but also to think well (Lawler & Salzman, 2013), a concept all too absent from the box-ticking compliance attitude which pervades modern business activity.

## Lonergan’s Concept of the Good

To understand the common good “one needs something in the way of a scheme, something that will suggest to one the great variety of questions connected with thinking about the human good” (Lonergan, 2010, pp. 494–495). Such a scheme will incorporate the self-reflexive decision-making process that is used to generate insight (see Fig. 1). For Lonergan, the structure of the human good is an open one, one that is big enough “to include both subject and object, to unite the subjective and objective, the individual and the social” (Lonergan, 2000, pp. 39–40).

Lonergan’s “good is concrete because it is what benefits, improves and enriches human living. It is what is good for individual persons, natural communities like family, and constructed communities like neighborhoods and nations” (Sauer et al., 2001, p. 61). Pursuing the common good necessitates thinking about how the various parts and their interrelationships can be maintained, corrected, and developed so that the whole community flourishes in a way that improves the well-being of its various parts and the individuals who ultimately make up those parts (Stebbins, 1997). As we move from the first level towards the third in Fig. 1, we, as virtuous persons, achieve a more comprehensive capacity to evaluate ethically the good that is attained at each level. As we engage in our daily activities, Lonergan helps us to illuminate, or make concrete, that our meaning of the good can be found at three interrelated stages which incorporate the hierarchy of: particular good; good of order; and value (McAleese, 2012). Schemes of recurring events that ensure provision of the human good break down, and private or individual good (particular good) takes the place of the common good. “So the good of order deteriorates” (Lonergan, 1972, p. 54). Particular goods, namely Lonergan’s first stage of the good, involve the desire for the satisfaction of vital needs such as food and shelter. At this level, the good is something that satisfies an individual desire or a personal interest. For example, this level of good is depicted in the capitalist model as the solitary pursuit of self-interested desire becoming the universal law of the market (McAleese, 2012; Melchin, 2005). However, while our desire for food, shelter, and income might be personal, its fulfillment depends on our participation in vast structures of cooperation comprising huge numbers of personnel engaging in complex patterns of interlocking tasks and roles (Melchin, 2005). The good of order comes about when we expand our focus from satisfying only our individual good(s) to satisfying the good that is found in social order. Consequently, while the first stage takes into account the needs and desires of individuals, the second stage of the human good focuses on cooperation. Accordingly, the good of order represents the coordination of human activity (Lonergan, 1992). As we progress from a focus on individual needs and desires from level 1 in Fig. 1, the good of order seeks its meaning from the reality of the individual within his/her socio-cultural world. Patterns of cooperation emerge, and “these patterns have their own routines and habits that are both technical and moral in nature” (McAleese, 2012, p. 96). Within these functions, individuals assume a range of obligations that are defined, not relative to any person’s needs or desires, but rather in terms of the cooperative scheme of the function and the function’s pattern of interaction with other activities both internal and external to the organization (Melchin, 2005). These recurring patterns of cooperation allow us to view the business organization as something beyond the concept of competing interests through a nexus of contractual obligations (McAleese, 2012), an alternative to Weber’s (1958) analogy of the ‘iron cage’ used to describe conventional management thinking and practice. How a group of people within an organization choose to cooperate depends on the insights each individual brings and shares in common with the others. As more insights are shared within the organizational community, a pattern of cooperation emerges to be represented by norms and values by which the community has chosen to abide. “The good expressed here asks whether the patterns of cooperation routinely occur to produce concretely particular goods to meet not just an individual’s needs, but the needs of many people time and time again” (McAleese, 2012, p. 97). Logic and meaning are embedded in the pattern of the cooperative process itself, a distinct level of moral meaning which is irreducible to mere desire (Melchin, 2005). The movement from the first to the second stage of human good is the progression of becoming a more enlightened and virtuous person, which, if we are attentive, we can observe within ourselves. Given that these patterns of cooperation and self-transcendence change to meet new and emerging needs of individuals, they are also, by necessity, dynamic. This is evidenced in the way some businesses respond to social and environmental crises. For example, do patterns of cooperation address sustainability and justice issues or do they ignore them?

Lonergan then moves his attention from the second stage of human good to the third. “When human beings are reflective and rational, particular goods, institutions and goods of order are inextricably bound to be considered, evaluated and criticized” (Lonergan, 2000, p. 39). Reflecting on the good of order and evaluating and criticizing it gives rise to the notion of value, namely asking the question, is it worthwhile? (Lonergan, 2000). The good, as value, calls for a critical evaluation of social orders within wider, more universal horizons of historical progress or decline. As a transcendental notion, value promotes the subject to full consciousness, directs him/her to his/her goals and provides the criteria for judging whether the goals are being reached (Ogbonnaya, 2013). “The key question guiding our operations of meaning is not simply the intelligibility of the social order (namely good of order), but… [the contributions of the institutions making up the social order] to the wider project of human living” (Melchin, 2007, p. 190). It is at this level that we see such values as dignity, care, and respect for others and the wider environment. Experiencing needs and desires (particular good) is a prerequisite for understanding patterns of cooperation (good of order). Paying attention to the cooperative schemes ensures the ongoing function of the good of order. Discerning whether the dynamism of these patterns of cooperation progress or decline the common good, is a judgment based on values (McAleese, 2012). Finally, decisions and actions taken are a culmination of the cognitive activities and are in accordance with the good. This method of self-discovery, a movement towards moral conversion, by way of the cognitive transcendental process, leads us to a sense of ethical agency, or communal responsibility, an end point representing Lonergan’s dynamic invariant structure of the human good. We discern and contribute to the human good by desiring it through our unrestricted notion of value, and then pursuing that desideratum faithfully through the dynamic cognitive structure of human knowing which underpins the EDM framework in Fig. 1. We are able to choose the good over self-satisfaction through grasping the reality of our lives and by appropriating the virtues of attentiveness, intelligence, reasonableness, and responsibility.

Lonergan’s dual dialectic of subject and community advances a normative dimension to EDM. His philosophy offers focus in this regard by providing a methodology to heighten awareness of the dialectic of the free responsible human person, pulled in opposing directions by desires, longings, and ambitions, struggling between the search for real self-transcendence and the pseudo version that views success, self-interest, wealth, and power as self-fulfillment (Cronin, 2006). What is the best way to live as a person in a community? How do we develop as free and responsible persons, knowing, deciding, and taking action in a responsible way for the common good? Those working in organizations are obliged to obey the rules and observe organizational norms and routines, not least because of the threat of sanctions. However, these are not moral obligations yet there is an expectation that individuals should act responsibly in this regard. Lonergan allows us to infuse responsibility into the scenario. At the fourth level of consciousness (Fig. 1), Lonergan (1972) asserts the importance of being responsible so that moral values cannot be reduced to other values, for example not doing something because it is against the rules. Being responsible recognizes that we are obliged to live and act in a society with others, and therefore, we have a responsibility to ourselves and to others. Being responsible is tantamount to deliberately choosing to act in accordance with the good where the good includes not only the object of desire but also the good of order and value as objects of rational choice. Lonergan’s cognitive structure and structure of the human good encourages a dynamic understanding of morals and virtue ethics through personal and communal horizons in which we seek answers to fundamental questions about life and collaborative solutions to advance the common good (Copeland, 1991). Such a philosophical anthropology attends to a fundamental and unwelcome consequence of capitalism, namely, the disconnect “between material problems and the deeper moral and spiritual values of humankind” (Wogaman, 1986, p. xi). Self-transcendence empowers us to avoid the dichotomy between knowing and doing and to imbue the community with meaning and values that promote the common good. A community will prosper when human beings espouse virtues to be attentive, be intelligent, and be reasonable and responsible without coercion. Envisioning business organizations as communities of persons engaging in cooperation is “more appropriate than seeing them as an aggregate of individuals united exclusively by contracts or [vested] interests” (Melé, 2012, p. 98).

## Conclusion

Against the backdrop of society’s grand challenges, in particular the COVID-19 pandemic, this paper makes a timely contribution to the extant business ethics literature by moving beyond traditional ethical methodologies which form the basis for corporate codes of ethics and standards, to embrace a Lonerganian decision-making framework which guides us as individuals to discover in ourselves the dynamic structure of our own cognitional and moral being. The timeless present of the extant business ethics narrative devotes insufficient attention to the role of the subject in the subject–object referent. Extant EDMs tend to emphasize obligations, rules, and criteria for judgment (Nunziato & Hill, 2019), whereas a Lonerganian perspective prioritizes thinking for ourselves as being indispensable to meaningful understanding, insights, judgment, and ethical action. Codes and universal standards can easily become static and dated when not grounded in the conscious operations of human subjects. When the good is judged in terms of the benefit to others then one moves towards self-transcendence and the altruism needed for ethical decision making and action to be promoted.

Similar to other seismic global events, COVID-19 could potentially transform how we see the world, the ways in which we think, and how we behave. Given that inattention, unreasonableness and irresponsibility have contributed to the COVID-19 crisis, economic, and societal advancement requires us to be more attentive, ask questions, look for the evidence, engage with insights, make judgments, and then act. This is Lonergan’s enduring contribution, namely the unfolding of that spirit of curiosity which wonders, inquires, and comes to understand, judge, and decide. By recognizing business ethics as a product of our own immanent and invariant operations of consciousness, we present a novel EDM perspective which proffers a new vantage point on developing ethical business culture and organizational strategy. This focus on individual decision-making agents’ engagement in self-appropriation articulates a normative transcendental standard which delivers the critical moral realism needed to address the grand challenges of our time.

Occasions have been few when the world has experienced such uncertainty yet known such opportunity for introspection to ignite Lonergan’s epistemology than is the case now during the COVID-enforced isolation and remoteness. Current circumstances afford us the space to engage with a recursive process which cultivates and incorporates virtues as indispensable normative qualities to direct our EDM within business. Be it in a virtual environment or otherwise, we are participants in the corporation, moving forward, to be guided by virtuous intent and obedient to the transcendental precepts. The moral and existential orientation of authentic obedience to these precepts is the intention, promotion, and active pursuit of the human good in common.

Lonergan himself would be the first to admit that none of this is easy. It takes perseverance, critical reflection and practice for us to become sensitive to the dynamics operating in our consciousness. Moreover, the personal challenge of sustaining intellectual and moral development and the seductive lure of competing ideologies present additional barriers to self-transcendence. When individuals and organizations make judgments and decisions distorted by egoism and group bias, as has been the case during the pandemic, the successful functioning of society is interrupted and a cycle of decline is triggered. However, notwithstanding these seemingly inherent challenges and limitations in the application of Lonergan’s philosophy, over time the persistent, cumulative, and potentially self-correcting processes of experiencing, understanding, critically reflecting, judging, and choosing help to surmount some of our own biases, the biases and errors of our culture and the biases inherited from our antecedents. Incorporating our novel EDM framework into an alternative corporate accountability mechanism, rather than relying on a codified system based on sanctions and enforcement, would nurture individual ethical responsibility. Accordingly, business leaders should direct their focus towards creating an environment and establishing a business ethos which encourages and promotes ongoing critical enquiry and reflexive thinking. Such enlightened questioning, we believe, would help promote a culture of individual responsibility within the corporate entity to advance the common good. To this end, future research could consider the application of our Lonerganian EDM framework in an empirical setting. This could be achieved by employing an abductive organizational case study approach.

In today’s society, where ongoing crises have left many of the most vulnerable to bear the health, economic, and ecologic burden of others’ hubris, greed, and denial and where COVID-19 is predicted to exacerbate existing inequalities (Kniffin et al., 2021), Lonergan’s teachings provide an alternative restorative human paradigm of business, portraying the corporation as a community of persons nurturing authentic ethical thought, decision making, and action. While COVID-19 is, first and foremost, a global health tragedy, it may also present a new dawn: one in which businesses have the opportunity to foster cognitive operations among their employees so that the ethical criteria of sustainability and self-transcendence pervade decision making. To this end, we trust that the message conveyed in our paper will contribute to and inspire ongoing conversations on repositioning business organizations to promote human flourishing**.**
